# Kidney ion handling genes and their interaction in blood pressure control

**DOI:** 10.1042/BSR20220977

**Published:** 2022-11-16

**Authors:** Caiyan An, Liuyi Yang, Tengfei Han, Huazhong Song, Zichao Li, Junjing Zhang, Kejin Zhang

**Affiliations:** 1 Foundational and Translational Medical Research Center, Department of Allergy and General Surgery, Hohhot First Hospital, Hohhot 010030, China; 2 Department of Pathophysiology, Basic Medicine College of Inner Mongolia Medical University, Hohhot 010050, China; 3 Department of Medical Imaging, The First Clinical Medical College of Inner Mongolia Medical University, Hohhot 010050, China; 4 Department of Biological Sciences, College of Life Science, Institute of Population and Health, Northwest University, Xi’an 710069, China

**Keywords:** blood pressure, Essensial hypertension, Functional study, Kidney ion handling genes, Kidney sodium reabsorption, protein-protein interactions

## Abstract

Hypertension affects 30% of adults and is the leading risk factor for cardiovascular disease. Kidney sodium reabsorption plays a vital role in the initial stage and development of essential hypertension. It has been extensively reported that the variants of kidney ion handling genes are associated to blood pressure, and clinical features of hypertension. However, the underlying mechanisms by which these variants alter protein function are rarely summarized. In addition, the variation of one single gene is often limited to induce a significant effect on blood pressure. In the past few decades, the influence by genes × genes (G × G) and/or genotype × environment (G × E) interactions on a given trait, for example, blood pressure, have been widely considered, especially in studies on polygenic genetic traits. In the present review, we discuss the progress in genetics studies on kidney ion handling genes, encoding Na^+^ channels (Na^+^-Cl^−^ cotransporter [NCC], Na-K-2Cl cotransporter [NKCC2], epithelial Na^+^ channels [ENaCs]), K^+^ channel (renal outer medullary potassium channel [ROMK]), and Cl^−^ channels (Pendrin, chloride voltage-gated channel Kb [CLC-Kb]), respectively, and their upstream kinases, WNKs and SGK1. We seek to clarify how these genes are involved in kidney sodium absorption and influence blood pressure, especially emphasizing the underlying mechanisms by which genetic variants alter protein functions and interaction in blood pressure regulation. The present review aims to enhance our understanding of the important role of kidney ion handling genes/channels in blood pressure control.

## Introduction

More than 1.1 billion people worldwide have hypertension [1,2], which is the main risk factor for stroke, coronary heart disease, kidney disease and other diseases, and responsible for estimated 7.8 million deaths worldwide in 2015 alone [3]. The pathogenesis of hypertension is complicated, including increased sympathetic nerve excitability, up-regulation of the renin-angiotensin-aldosterone system (RAAS) and renal sodium reabsorption, vascular damage, immune system dysfunction, and inflammation. Among those, renal sodium reabsorption plays an important role in blood pressure regulation; in fact, RAAS also involves renal sodium reabsorption through stimulating the release of aldosterone [4].

Earlier, people have realized that abnormal renal function is closely related to one’s blood pressure [5,6]. Over 4500 years ago in China, the *Yellow Emperor’s Classic of Internal Medicine* has suggested that ‘the kidneys pass on the diseases to the heart’ [7]. However, the mechanism by which the kidney regulates blood pressure was still not fully clarified [8], until the 1960s, Guyton’s ‘pressure natriuresis relationship’ model first explained the underlying mechanism by which renal sodium excretion is closely related to long-term blood pressure regulation [9–12]. Increased renal sodium reabsorption leads to enhanced water reabsorption in the kidneys, a subsequent increase in blood volume and the venous blood flowing back to the heart, and finally leads to an increase in blood pressure [4,12]. The evidence of blood pressure travels with the kidney in Milan rats had indicated that the process of renal sodium reabsorption is determined by genetic background, which is sufficient to alter blood pressure in the recipients [13].

**Figure 1:**
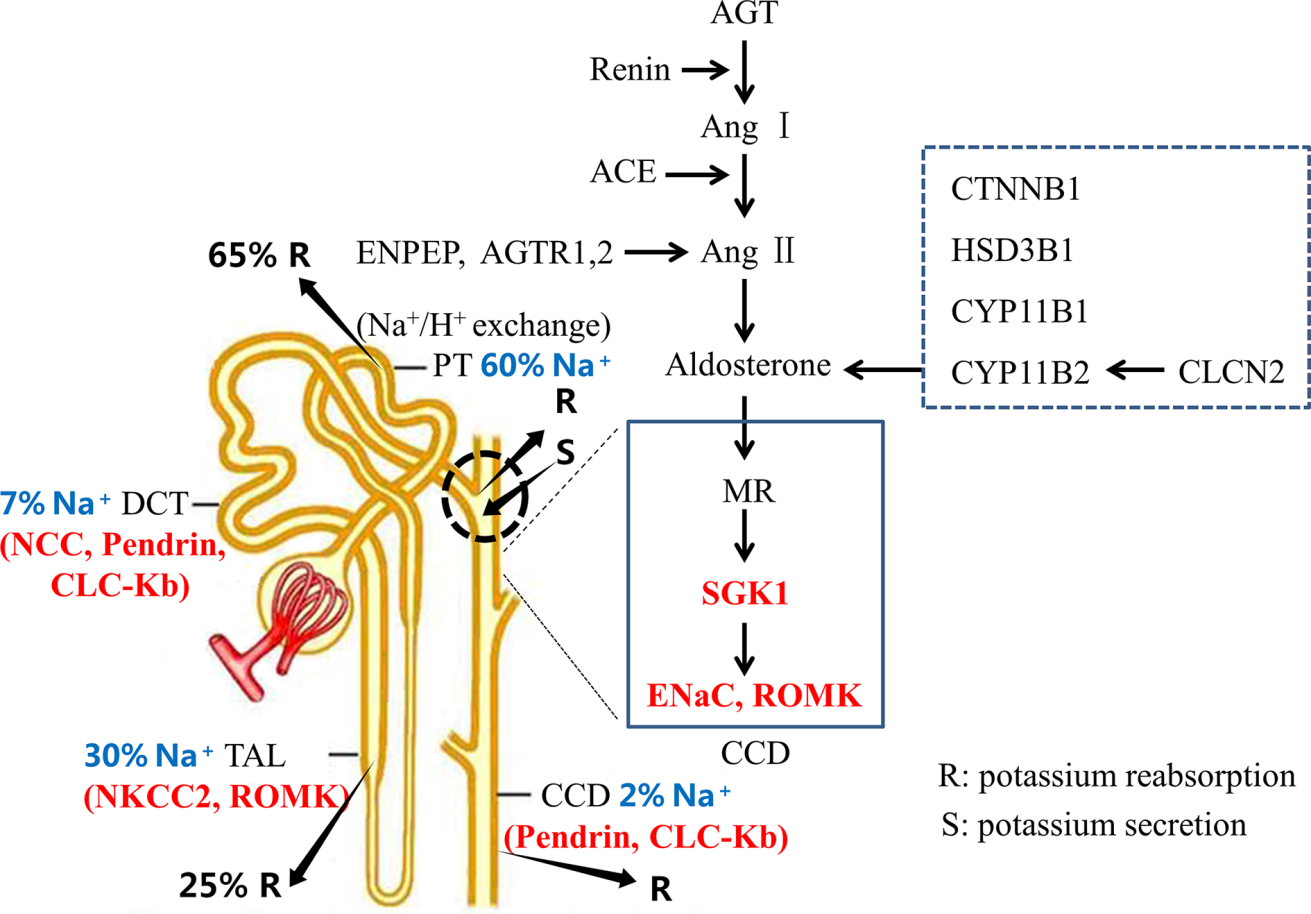
Renal sodium reabsorption-related genes involved in blood pressure regulation Channels or kinases discussed in the present review were marked in red. The relative amounts of reabsorption (for sodium and potassium) and secretion (for potassium) in the different parts of kidney were shown as follows: PT (60%), TAL (30%), DCT (7%), and CCD (2%) for sodium reabsorption; PT (65%), and TAL (25%) for potassium reabsorption; and potassium secretion is accomplished mainly in the part of DCT. Abbreviations: PT, proximal tubule; TAL, thick ascending limb; DCT, distal convoluted tubule; CCD, cortical collecting duct.

Following recent breakthroughs in genotyping, and sequencing numerous genes and/or their variations related to hypertension and other relative disorders have been identified. For instance, summary statistics of the NHGRI-EBI Catalog of human genome-wide association studies (GWAS Catalog, https://www.ebi.ac.uk/gwas/home) records that about 733 genetic genes/variants in 90 studies associate to persistently high systemic arterial blood pressure. Up to April 2022, in PubMed database (https://pubmed.ncbi.nlm.nih.gov/) more than 56 studies discuss the relationship between genetic variants of genes related to renal sodium reabsorption and blood pressure regulation. These identified renal sodium reabsorption-related genes involved in blood pressure regulation included: (i) Kidney ion handling genes, encoding Na^+^-Cl^−^ cotransporter (NCC), Na-K-2Cl cotransporter (NKCC2), sodium channels (ENaC), renal outer medullary potassium channel (ROMK), Pendrin, chloride channels (CLC-Kb), respectively, and their upstream kinases, WNKs and SGK1, etc.; and (ii) RAAS genes, including AGT, REN, Ang I, Ang II, ACE, CYP11B2, and aldosterone etc., which participate in the kidney sodium reabsorption via the aldosterone/ENaC pathway (Figure 1 and Supplementary Table S1). Genetic variants leading to the alteration of renal sodium reabsorption are significantly associated to people’s blood pressure, and then responsible for clinical features of hypertension [14]. However, most studies usually focused on one or two kidney sodium reabsorption-related genes, which participate in blood pressure regulation, rather than provided a panel of kidney ion handling genes in blood pressure control. Meanwhile, only a few studies (i.e., Ashley et al. [15] summarized the partial mechanisms of ENaCs) elaborated detailed mechanisms by which the variants in kidney ion handling genes change the function of proteins and the interaction of G × G in blood pressure regulation.

The kidney handles a wide variety of ions including sodium, potassium, chloride, magnesium, calcium, and others to influence blood pressure. However, in the presnt review, we focused on the latest genetic studies of kidney sodium, potassium, and chloride handling genes, especially in the thick ascending limb of Henle’s loop (TALHL), distal convoluted tubule (DCT), and cortical collecting duct (CCD), encoding Na^+^ channels (NCC, NKCC2, ENaCs), K^+^ channel (ROMK), and Cl^−^ channels (Pendrin, CLC-Kb), respectively, and their upstream kinases, WNKs and SGK1, and clarified how they were involved in kidney sodium absorption and influence blood pressure, and elaborated the underlying mechanisms of these genes variations altering protein functions and their interaction in blood pressure regulation, which may provide an opportunity to understand the relationship between genetic background of renal physiology and blood pressure regulation, and individual susceptibility to hypertension [16], and further emphasized the significance of renal sodium reabsorption in blood pressure control.

## Genetic variants of ion channels in the kidney

The process of reabsorption in the kidney is, through its specialized ion channels (e.g., Sodium channels—ENaC; Potassium channels—ROMK; and Chloride channels—CLCs, etc.), the nephron removing water and solutes from the tubular fluid and returning them to the circulating blood, and ensuring appropriate electrolyte homeostasis. Given the blood volume closely influenced by renal sodium reabsorption, genetic variants, and mutations of ion channels involving the process of this reabsorption are being paid attention to by researchers and clinical doctors. Two decades of genetics studies are also indicated that these genetic backgrounds may be responsible for individuals’ blood pressure through altering the process of reabsorption on ion channel(s).

### Genetic variants of Na^+^ channels

Na^+^ channels in the TAL, DCT, and CCD of kidney are mainly composed of ENaCs, NCC, and NKCC2. Human ENaCs, as the end effector of adrenocortical hormones and excitable epithelial Na^+^ channel, has four subunits: α, β, γ, and δ encoded by *SCNN1A*, *SCNN1B, SCNN1G*, and *SCNN1D*, respectively [17], which are mainly located in connecting tubule (CNT) and collecting ducts (CD) and responsible for the rate-limiting reabsorption of Na^+^ in the kidney, and plays an important role in maintaining homeostasis of extracellular fluid volume, blood pressure, and sodium. Gain-of-function mutations in ENaC subunits β and γ cause a rare hereditary hypertension, Liddle syndrome. Conversely, loss-of-function mutations in ENaC result in a kind of hereditary hypotension, pseudohypoaldosteronism type 1 (PHA 1) [15]. Association studies also showed that ENaC variants (e.g., p.T663A of *SCNN1A* [18], p.T594M of *SCNN1B* [19], and c.G(-173)A of *SCNN1G* [20]) are closely related to hypertension in the general population (Supplementary Table S3).

Functional studies showed that variants of the *SCNN1A*, *SCNN1B*, and *SCNN1G* genes mainly change the function of Na^+^ channels through four mechanisms: **(i) Influence the surface expression of ENaC channel** (e.g., p.C618F [21], p.A334T [22], p.V481M [22], p.C479R [23], and p.A663T [21,24] of *SCNN1A*, and c.G(-173)A [20] of *SCNN1G*. For instance, Tong et al. [21] found that αhENaC variants p.C618F and p.A663T increased the number of apical membrane αhENaC, thereby increasing the activity of αhENaC above 3.3 times and 1.6 times, respectively. **(ii) Modulate the open probability of channels (Po).** The open probability of ion channels is positively correlated with channel activity. With the enhancement of channel open probability, the channel activity increases. Some variants (e.g., p.W493R of *SCNN1A* [25], p.R564 stop mutation of *SCNN1B* [26], and p.L511Q of *SCNN1G* [27]) have been reported to change the open probability of channels. Of them, *SCNN1G* p.L511Q [27] increased the open probability of the channel by four-fold compared with wild-type cells; **(iii) Affect the proteolytic cleavage of ENaC protein.** The activity of ENaC is also affected by the state of proteolytic cleavage. The channel product after proteolytic cleavage has a high-channel open probability due to the release of an inhibitory tract in ENaC protein, while the uncleavage channel is in an inactive state [28–31]. Knight et al. [28] found ENaC gene mutation not only changes surface expression of the channel but also affects the activity of the ENaC channel by changing the proteolytic cleavage state of the channel protein. Kota et al. [32] demonstrated that an alternative splicing variant of αENaC (αΔ34–82-ENaC) significantly reduces the channel activity, which is mainly due to the increase in ‘uncleaved, near-silence ENaC’; **(iv) Alter the Na^+^ self-inhibition on ENaC channels**, e.g., p.A334T [22], p.R476W [22], p.V481M [22], p.H239R [33], p.H239D [33], and p.H239C [33] of *SCNN1A*; p.γH239R [33], p.γH239D [33], p.γH239C [33], p.L511Q [27] of *SCNN1G*. ENaC selectively allows extracellular sodium ions to enter endothelial cells, and at the same time, elevated extracellular sodium ions have a certain inhibitory effect on ENaC channels, which is referred to as Na^+^ self-inhibition [15,33–35]. Na^+^ self-inhibition enables the distal nephron to control Na^+^ reabsorption based on the urinary Na^+^ concentrations and maintains sodium homeostasis in body fluid [15]. Many studies have found that ENaC variants affect the channel function by altering Na^+^ self-inhibition [30,36–38]. For example, Rauh et al. [25] reported that compared with wild-type cells, αENaC W493R mutant cell increased the amiloride-sensitive whole-cell current by about four-fold, partially by reducing the inhibitory effect of extracellular sodium ions on ENaC, thereby increasing the activity of ENaC channels.

**Figure 2:**
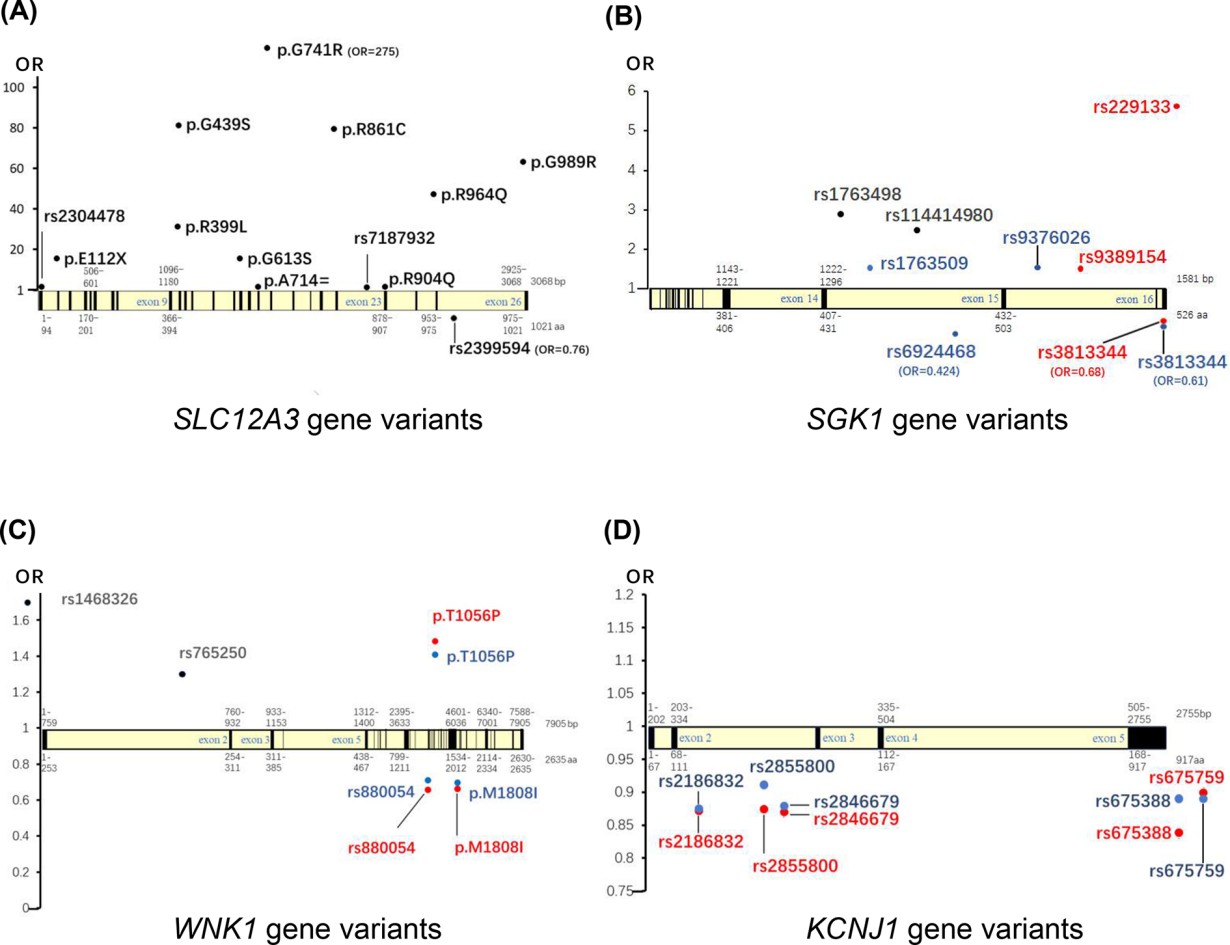
Variants of kidney sodium reabsorption-related genes and their effects on SBP (red), DBP (blue), and hypertension (black) (**A**) *SLC12A3* gene; (**B**) *SGK1* gene; (**C**) *WNK1* gene; (**D**) *KCNJ1* gene. The detailed information are shown in Supplementary Table S3.

Besides ENaC, two cotransporters including NCC and NKCC2 are also involved in kidney sodium reabsorption, and exert vital roles in blood pressure regulation. For NCC, its encoding gene *SLC12A3* is specifically expressed in the apical membrane of the DCT cells of kidney, whose genetic variants and mutations (Figure 2A and Supplementary Table S3) show closely relationship to individual’s blood pressure and/or hypertension [39–41]. *SLC12A3* gene mutations cause the functional changes of NCC protein through modifing NCC’s expression (e.g., *SLC12A3* p.S186F [42]), localization (e.g., NCC_1/2_ variants [43]), and activity (e.g., *SLC12A3* p.Arg919Cys [44]) in membrane of cells. Moreover, several studies have also found that NCC transporters carrying different mutants have a different affinity for thiazide diuretics. For instance, in 2006, Moreno et al. [45] defined NCC transporter TM (transmembrane domain) 8–12 as a residue domain with high thiazide affinity; in 2007, Vormfelde et al. [46] found that subjects with NCC G264A mutants had greater diuretic response to diuretics; in 2010, an *in-vitro* validation study with Xenopus oocytes found that mutations in specific amino acids located within TM8-12 were the main cause of thiazide affinity differences between NCC transporters containing different mutants [47].

For NKCC2 encoding gene *SLC12A1*, it is specifically mainly expressed in the apical membrane of the TALHL and responsible for nearly 24% of kidney sodium reabsorption [48]. Therefore, NKCC2 is one transporter with the strongest renal sodium reabsorption capacity and even subtle changes in the activity of NKCC2 can significantly change kidney sodium reabsorption [48]. As listed in Supplementary Table S3, mutations of *SLC12A1* were observed having strong association to blood pressure and hypertension in the general population [49,50]. Functional verification showed that variants of *SLC12A1* change the protein function through effecting the surface expression (e.g., p.R302W and p.L505V [51]), location (e.g., p.R302W and p.L505V [51]), and activation (e.g., p.R298W, p.P344L, p.L501V, p.N395S, p.P565H, and p.Y1066C [42]) of NKCC2. Some studies have also found that the current targeted medicines to NKCC2 have differential action depending on the underlying mutation in NKCC2. For example, NKCC2 with different mutants have different affinities for a loop diuretic (bumetanil) [46].

### Genetic variants of K^+^ channel

The ROMK (Kir1.1) is a kind of K^+^ channel, encoded by the *KCNJ1* gene. ROMK is mainly expressed in two areas of kidney: CCD and TALHL. ROMK strictly regulates the secretion of potassium in CCD and at the same time controls the potassium cycle in TALHL. Potassium plays an important role in the regulation of blood pressure. Diet potassium intake, serum potassium, and urinary potassium are all negatively associated with blood pressure [52,53], and for example, low potassium diets could lead to salt-sensitive hypertension [54]. Potassium regulates blood pressure mainly via affecting kidney sodium reabsorption, vasodilation, baroreflex sensitivity to catecholamine and angiotensin II, and other mechanisms [52]. Specifically, how does potassium channel affect renal sodium reabsorption? Taking ROMK as an example, (i) in CCD cells, ROMK is the driving force for ENaC; (ii) in TAL, ROMK is the driving force for NKCC2 as well [55]. The activity of ROMK channel decreases the concentration of intracellular K^+^ (that is a decrease in intracellular cation concentration). Therefore, maintenance of intracellular charge homeostasis requires ENaC and NKCC2 reabsorb more cation (Na^+^, etc.) into the cells [55], ultimately causing the increase in kidney sodium reabsorption and subsequent hypertension. ROMK inhibitors have been used as novel diuretic targets for the treatment of hypertension and heart failure [56].

The *KCNJ1* gene was previously reported to associate with blood pressure. For instance, loss-of-function mutations of the *KCNJ1* gene cause Barter syndrome type II characterized by kidney salt wasting, hypotension, and mild hypokalemia. And Ji et al. [49] determined that multiple variants in the *KCNJ1* gene caused a decrease in blood pressure in 3125 individuals and 292 Gitelman or Bartter syndrome patients in the Framingham Heart Study in the United States. Additionally, p.E151K [57], p.Y314C [58], p.T191N [58], p.E284Q [58], rs675759 [59], rs675388 [59], rs2846679 [59], rs2855800 [59], and rs2186832 [59] in the *KCNJ1* gene are observed to associate with blood pressure or hypertension (Figure 2D and Supplementary Table S3). Researchers have previously verified that the *KCNJ1* gene variation affect the function of ROMK channel through affecting the surface expression and the activity (e.g., p.R193P, p.H251Y, and p.T313FS), and confering a gain in regulated-inhibitory gating (p.P166S and p.R169H) of ROMK [60].

### Genetic variants of Cl^−^ channels

Extracellular fluid volume is determined by NaCl, but not by Na^+^ only; therefore, Cl^−^ transporter/channels, such as Pendrin and CLC-Kb, also play important roles in blood pressure control [61]. Pendrin protein encoded by the *SLC26A4* gene (also *PDS*) is a transmembrane chloride/anion transporter and mediates the secretion of bicarbonate and the reabsorption of chloride, and mainly expressed in the thyroid [62], inner ear [63,64], and kidney [65]. The expression of pendrin in the kidney is regional, and mainly distributed on the apical membrane of the intercalated cells in the posterior segment of DCT, CCD, and CNT [66]. Wall et al. [67] reported that pendrin disruption not only impairs the secretion of HCO^−^ but also totally abolished Cl^−^ reabsorption, and pointed out that the Cl^−^ transport regulated by pendrin is the most important Cl^−^ reabsorption pathway in the collection system. Pendrin gene ablation (SLC26A4^−/−^) has been reported to associate with reduced renal sodium reabsorption and blood pressure and resistant to hypertension caused by aldosterone *in vivo* [68,69].

Association analyses have also confirmed that *SLC26A4* gene variants are related to SBP and DBP [70]. In functional studies, pendrin P70L, P301L, F667C [71], E29Q, V88I/R409H, G424D, T485R [72], V239D, G334V/X335, I487Y/FSX39 [73], and V510D [74] are found to be the reduction or loss of function variants, whereas pendrin V88I and G740S exhibit a gain of function [71]. To date pendrin variants identified by functional studies have been well summarized in the review of Dossena et al. [75], which pointed out that the involvement of a charged amino acid of pendrin is not always sufficient to induce a detrimental effect on the ion transport such as D266N and K369E.

Another important chloride channel is CLC-Kb encoded by the *CLCNKB* gene. The ClC-Kb channel and its accessory subunit barttin (encoded by the *BSND* gene) are expressed in Henle’s loop, DCT and CCD of the kidney, and their function is to help the reabsorption of chloride, and the maintenance of urine concentration [76]. Loss-of-function mutations in *CLCNKB* cause Barter syndrome type III, while gain-of-function mutations in *CLCNKA* and *CLCNKB* can cause rare salt-sensitive hypertension [77]. Accumulating evidences demonstrated that the *CLCNKB* gene variants, such as p.S12A, p.E192Ter [78], p.T481S [79,80], p.R27L [81], rs5253, and rs2275166 [82], are significantly associated with hypertension or blood pressure levels. Functional verification confirm that *CLCNKB* gene mutations affect the function of CLC-Kb channel through increasing the activity (e.g., p.T481S [79,83]), or reducing the activity of CLC-Kb channel (e.g., p.G167V [84], p.A242E [84], p.R351W [85], p.R30X [85], and p.A210V [85]).

### Two important kinases regulating kidney ion channels

With-no-lysine (WNK) and SGK1 are two important kinases regulating kidney ion channels, and hence involved in kidney sodium reabsorption and hypertension. WNK is a serine/threonine protein kinase, and four members of the WNKs family (WNK1–4) have been identified in mammals (Supplementary Table S2). WNKs are widely expressed in human organs and tissues. Except for WNK2, all the other WNKs are expressed in kidney [86]. Alternatively spliced transcript variants encoding different isoforms of WNK1–4 have been reported. The existence of these variants greatly enriched the functions of WNKs [87]. The best-studied variant of WNKs for hypertension is the kidney-specific WNK1 (KS-WNK1), a variant of full-length WNK1 (L-WNK1). KS-WNK1 is very specifically expressed in kidney, and antagonizes the function of L-WNK1 in blood pressure regulation [87].

There are interactions between WNKs as follows. **(i) WNK1 and WNK3 exist compensation effects.** In mice lacking WNK3, no obvious salt consumption phenotype was observed for compensatory up-regulation of WNK1/SPAK axis [88]; **(ii) WNK1/WNK3 and WNK4 inhibit each other.** In addition to act alone, WNK1 can regulate renal sodium handling genes by inhibiting WNK4 [89]. WNK3 carboxy-terminal domain reduced the WNK4 kinase domain affinity for chloride, which is important for WNK4 to regulate kidney ion channels [90]. Meanwhile, WNK4 antagonizes effects of WNK1 and WNK3 on NCC [91]; **(iii) KS-WNK1 inhibits the activity of L-WNK1/WNK3.** Based on observations in genetically inactivated mice of two WNK1 isoforms, the effect of activating L-WNK1 exceeds that of KS-WNK1 [92].

WNKs are extremely important kinases that connect angiotensin II, aldosterone, and renal sodium and potassium transporters. Mutations of WNK1 and WNK4 cause Gordon syndrome (pseudohypoaldosteronism type 2, PHA 2) accompanied by hypertension, elevated serum potassium, and acidosis [93]. Lalioti et al. [94] found that mice carrying PHA2 WNK4 mutant transgene have higher blood pressure than wild-type mice. In 2007, yang et al. [95] carried out a functional study *in vivo* and generated Wnk4^D561A/+^ knockin mice. They found this knockin mice increased apical expression of phosphorylated NCC protein in the DCTs through activation of the OSR1/SPAK-NCC phosphorylation cascade, and showed the phenotypes of hyperkalemia and hypertension.

Except for WNK2, the other members of WNKs family have been reported to be associated with blood pressure or hypertension. A lot of blood pressure-associated polymorphisms of WNKs have been identified, including rs1468326 [96], rs765250 [97], rs880054 [98,99], rs956868 [98], and rs12828016 [98,99] of the *WNK1* gene (Figure 2C and Supplementary Table S3); and p.Ala589Ser [100] of the *WNK4* gene.

The other important kinase in blood pressure control is a serum- and glucocorticoid-regulated kinase 1 (SGK1), which has at least three isoforms: SGK1 (iso-1), and its two N-terminal variant subtypes Sgk1_i2 (iso-2) and Sgk1_i3 (iso-3). Iso-2 and iso-3 are more stable than iso-1; Therefore, they are considered to be the main players for the role of SGK1 in hypertension [101–103]. SGK1 is highly expressed in renal tubules and plays an important role in sodium and potassium homeostasis and blood pressure regulation via activating ENaC [104–107]. In addition, SGK1 regulates the expression of NKCCs, NCC and NHE3 [108–113]. Evidences from experiments *in vivo* certified that high sodium intake up-regulates SGK1/ENaC pathway, leading to salt-sensitive hypertension [114]. In contrary, SGK1 deficiency prevents the occurrence of hypertension caused by a high-fat/high-fructose diet [104].

Some SNPs in the *SGK1* gene have been demonstrated to relate to blood pressure or hypertension, including rs1057293, rs1743966 [115–117], rs2758151 [118,119], rs9402571 [118], rs9376026, rs9389154, rs1763509, rs9376026, rs3813344 [120], rs1763498, rs114414980, rs229133, and rs6924468 [121] (Figure 2B and Supplementary Table S3). Zhang’s gene-based analyses declared that *SGK1* gene was associated with risk of hypertension (*P*=7.4 × 10^−3^) in the Chinese Han population [121].

SGK1 participates in the regulation of hypertension mainly through ENaC. *In-vitro* study demonstrate that iso-2 is preferentially localized to the plasma membrane, and can better stimulate ENaC compared with wild-type SGK1 [103]. In addition, iso-3 also dramatically enhanced ENaC activity through increasing the number of cleaved ENaC protein [102].

Taken together, accumulating evidences clarified that kidney sodium reabsorption-related genes did exert a vital role in blood pressure control. However, one gene’ function is limited to induce a significant effect on blood pressure. In the past few decades, the influence from the interactions of genes × genes (G × G) and/or genotype × environment (G × E) on a given trait (e.g., blood pressure) [122] have been widely considered, especially for polygenic genetic traits’ studies. In the following of the present review, we will discuss the interaction of G × G in blood pressure control.

## Interaction of genes × genes (G × G) related-renal sodium reabsorption in blood pressure regulation

### Compensation between NCC and other ion channels (Pendrin/ENaC)

Pendrin is a Cl^−^/HCO_3_^−^ transporter, and NCC is a Na^+^-Cl^−^ cotransporter, both of which play an important role in renal sodium reabsorption. When pendrin and NCC were mutated separately, the renal function of mice including sodium chloride excretion, urine output, and blood urea nitrogen were comparable with wild-type mice. However, when pendrin and NCC were double knocked out in mice, and they showed volume depletion or hypotension under salt restriction conditions [123]. Therefore, compensation exists between NCC and Pendrin in regulating renal function and blood pressure. For instance, NCC gene knockout mice do not have a significant salt loss for up-regulated Pendrin and ENaCs making up for NCC deletion [124]. Similarly, down-regulation of Pendrin in Carbonic Anhydrase II (CAII) knockout mice did not cause significant salt wasting because of compensatory up-regulation of NCC, but they showed severe salt wasting after NCC was inactivated or inhibited [125]. In future, targeted inhibition of both NCC and pendrin will provide a strong diuretic regimen for the treatment of hypertension.

Similar to NCC and Pendrin, compensation also exists between NCC and ENaCs, and their interaction maintains the homeostasis of systemic blood pressure. For example, it is generally believed that NCC activation causes hypertension. However, the result observed in KS-WNK1-KO mice is that NCC activity is significantly enhanced, but systemic blood pressure only showed a slight increase, and failed to cause hypertension, which is related to decreased expression of ENaC [126]. Conversely, genetic inactivation of NCC in mice indeed enhances the expression of ENaC and the absent effect of NCC phosphorylation in SPAK-KO mice is compensated by enhanced expression and function of ENaC [127,128]. In addition, NCC and ENaCs are coexpressed in DCT2 cells and a study confirmed that NCC interacts with ENaCα and ENaCγ subunits probably through directly binding [129].

Taken together, there are strong compensatory effects between NCC and Pendrin/ENaCs. When NCC or Pendrin/ENaC is knocked out or malfunction, the expression or activity of another protein will compensatively increase to maintain the homeostasis of individual’s salt wasting, urine output maintains, and blood pressure. However, NCC and ENaCs, or NCC and pendrin were both knocked out or inactivited, exceeding the compensatory ability between genes, which will lead to serious salt wasting or blood pressure variation.

### Pendrin working in tandem with ENaCs

Pendrin and ENaCs are expressed in aldosterone-sensitive kidney areas, mainly including DCT, CNT, and CCD. Previous studies showed that Pendrin works in tandem with ENaC, while NCC works alone [124]. In animal models, the up- or down-regulations of ENaCs and Pendrin showed to be highly consistent. For example, the abundances of ENaC and pendrin both increase with aldosterone and aldosterone analogs [130,131], dietary NaCl restriction [67,130], and Cl^−^ restriction alone [132–135]. Meanwhile, the abundance and activity of ENaC are reduced in pendrin-null mice, ultimately leading to a decrease in natriuresis and chloriuresis and blood pressure [68].

Pech et al. [136] showed that Pendrin gene ablation reduces ENaC-regulated renal sodium reabsorption by altering subunit abundance, subcellular distribution, and channel open probability of ENaCs in wild-type mice. However, how do Pendrin and ENaCs interact with each other? This has become the focus of attention of scientists, because ENaC and Pendrin are expressed in different types of cells in the kidney though their abundances show consistency in many animal models. ENaC is mainly expressed in the apical plasma membrane of epithelia of CNT and CD [137]. Nevertheless, Pendrin is mainly expressed on the apical membrane of intercalated cells in renal CCD, CNT, and DCT. Therefore, it is impossible for them to influence each other through direct protein–protein interaction. How they interact with each other remains to be studied in future.

### WNKs regulate Na^+^ and K^+^ channels in the kidney

WNKs protein and isoforms are composed of WNK1–4, and KS-WNK1 etc. Except for WNK2, all of the others are expressed in the kidney. As the difference of WNKs in structure, their regulatory functions for renal ion channels also vary.

#### Regulation of NCC and NKCC2 by WNKs

**Figure 3:**
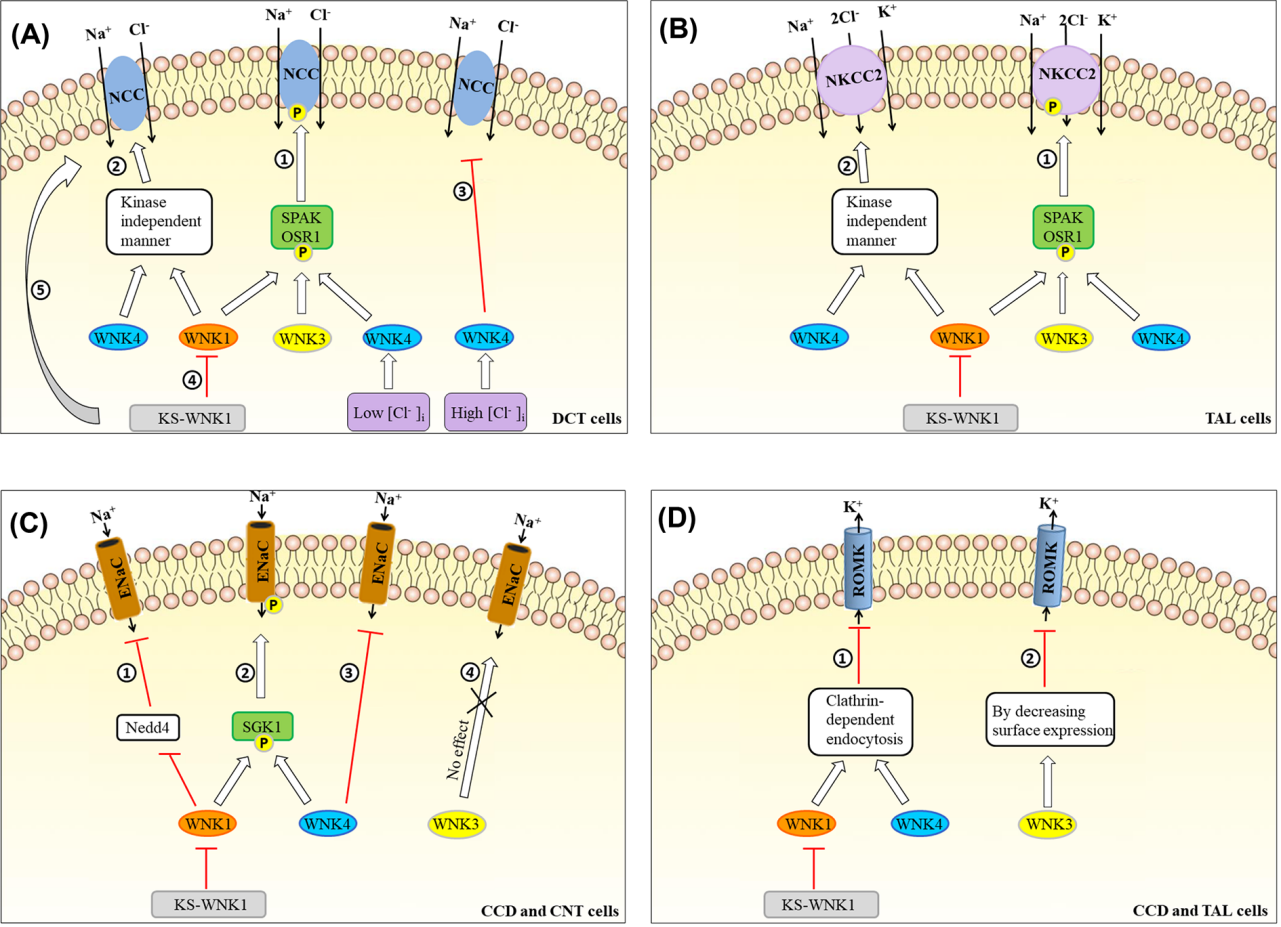
WNKs regulate Na^+^ and K^+^ channels in the kidney WNKs regulate NCC (**A**), NKCC2 (**B**), ENaC (**C**), and ROMK (**D**).

There are many regulatory mechanisms whereby WNKs regulate the function of NCC and NKCC2 (Figure 3A,B). First, both NCC and NKCC2 belong to the solute carrier family 12 (SLC 12), and WNK1 and WNK4 up-regulates NCC and NKCC2 in the same way and mainly via two mechanisms: (i) kinase activity-dependent (WNKs-SPAK/OSR1-NCC/NKCC2 pathway) and (ii) kinase activity-independent manners [138,139]. WNKs change activities of NCC and NKCC2 mainly in a kinase activity-dependent manner. SPAK/OSR1 are direct substrates of WNK1 and WNK4 [140,141]. For instance, Richardson et al. [139] reported that WNK1 phosphorylates and activates SPAK/OSR1, which further phosphorylates and activates NCC in HEK cells. And Castaneda-Bueno et al. [142,143] reported that WNK4-deficient mice showed decreased NCC phosphorylation and manifested hypotension. Differently, WNKs change surface expression of NCC and NKCC2 mainly through a kinase activity-independent manner (Figure 3A,B; **pathways 1 and 2**) [144,145]. Some reports showed that WNK1 up-regulates NCC via abolishing the inhibitory role of WNK4 on NCC [89].

Second, WNK3 increases NCC and NKCC2 through SPAK pathway (Figure 3A,B; **pathway 1**). WNK3 is expressed in DCT of the kidney, and its carboxyl-terminal can activate NCC and is one of the important members of up-regulating NCC [146]. Studies showed that wild-type WNK3 enhances membrane surface expression of NCC, mainly through phosphorylation of SPAK [146]; however, WNK3 with no kinase activity inhibits the expression of NCC [147]. Similar to NCC, WNK3 promotes the phosphorylation of NKCC2 by activating the SPAK pathway and mediates the chloride induction of TAL cells [146]. Although there is conclusive evidence that WNK3 is a stimulator of NKCC2 activity *in vitro*, the overall effect of this kinase on NKCC2 *in vivo* seems to be limited.

Third, the regulation of WNK4 on NCC is complicated, and besides the above positive regulation, WNK4 also plays an inhibitory role on NCC (Figure 3A; **pathway 3**). WNK4 has been reported to directly inhibit NCC and is a strong inhibitor of NCC, which deletion causes overactivity of NCC and thus hypertension [89,148]. Moreover, WNK4 inhibits the function of NCC by decreasing cellular surface expression of NCC, but not by reducing the transport capacity of NCC transporter. Gamba et al. [138] found that the concentration of Cl^−^ plays a vital role in the conversion of positive and negative regulations of WNK4 on NCC. They confirmed that WNK4 is a chloride-sensitive kinase, and its effect on the SPAK-NCC pathway is regulated by Cl^−^ concentration. Under low-chlorine conditions, WNK4 is automatically phosphorylated through a SPAK-dependent mechanism to activate NCC and play a positive regulatory role; Under high-chlorine conditions, WNK4 cannot be automatically phosphorylated due to its significant negative effects on WNK1 and WNK3, and inhibits NCC, plays as a negative regulator. NKCC2 is regulated by WNK4 via SPAK/OSR1 in a kinase activity-dependent manner [144].

Fourth, previous studies showed KS-WNK1 antagonizes the ability of L-WNK1 to inhibit NCC and NKCC2 (Figure 3A; **pathway 4**) [126,149]. KS-WNK1 and WNK1 form a dynamic balance and fine regulate NCC. However, recent studies certified that the KS-WNK1 isoform is a powerful activator of NCC as well, in which KS-WNK1 may activate an endogenous SPAK-dependent pathway that is not affected by L-WNK1 (Figure 3A; **pathway 5**) [150].

#### Regulation of ENaC by WNKs

WNK1 activates ENaC via two pathways: (i) WNK1-Nedd4-ENaC pathway (Figure 3C; **pathway 1**); and (ii) WNK1-SGK1-ENaC pathway (Figure 3C; **pathway 2**) [151,152]. WNK4 up-regulates or down-regulates the protein level of ENaC, respectively, by two different pathways: (i) WNK4 increases the expression of ENaC protein on the plasma membrane via phosphorylating SGK1 (Figure 3C; **pathway 2**) [86]; and (ii) WNK4 down-regulates ENaC level in a kinase-dependent manner probably involved in the regulation of Nedd4 (Figure 3C; **pathway 3**) [153]. However, WNK3 has almost no effect on the activity of ENaC (Figure 3C; **pathway 4**) [154].

#### Regulation of ROMK by WNKs

First, WNK1 and WNK4 inhibit ROMK via enhancing clathrin-dependent endocytosis, and the C terminus of ROMK plays an important role in the inhibitory effect (Figure 3D; **pathway 1**) [155]. Second, WNK3 inhibits the activity of ROMK by varying the membrane surface expression of ROMK instead of varying the conductance or opening probability of ROMK1 channel (Figure 3D; **pathway 2**). Third, KS-WNK1 has no kinase activity and cannot block ROMK directly. It indirectly activates ROMK by antagonizing the inhibition of L-WNK1 on ROMK [156]. Cheng et al. [157] found that ROMK-regulated K secretion significantly decreased in KS-WNK1 knockout mice, confirming that KS-WNK1 does indirectly activate ROMK *in vivo*.

### SGK1 up-regulates Na^+^, K^+^, and Cl^−^ channels in the kidney

SGK1 exerts a vital role in regulating Na^+^, K^+^, and Cl^−^ channels in the kidney. Among them, the regulation of SGK1 on ENaC, ROMK, and CLC-Kb has been well summarized in the review of Valinsky et al. [158] In addition, SGK1 can also up-regulate NKCC [112] and NCC [111], and enhance the sodium reabsorption in the renal tubules, resulting in an increase in extracellular fluid volume and blood pressure.

## Conclusions and perspectives

Hypertension affects 30% of adults and is the leading risk factor for major cardiovascular events such as heart attack and stroke, chronic kidney disease, and heart failure [159]. Kidney ion handling genes participate in kidney sodium reabsorption and play a vital role in the initial stage and development of essential hypertension. The past few decades have seen remarkable progress in the studies on kidney ion handling genes and blood pressure control, and consequently antihypertensive medicines targeting kidney ion handling genes (such as thiazide diuretics for NCC) have been developed and available for clinical treatment. Nevertheless, in clinical practice hypertensive patients caused by up-regulation of kidney sodium reabsorption have yet to be identified and separated from patients with hypertension due to other causes, such as increased sympathetic nerve excitability and immune activity, up-regulation of RAAS, and vascular damage. The following two key points are especially worthy of noting. The first is that the clinical translation of molecular genetic findings is relatively lagging. Most hypertensive patients have not been tested for susceptibility genes or drug sensitivity before treatment and their treatments are consequently largely empiric though there are many evidences that the current medicines (e.g., thiazides, and loop diuretics) have differential action depending on the different mutation in the transporter [45–47]. In the future, promoting clinical translation and developing test kits based on molecular genetic findings should be the top priority of our work. Also, the research progress in the genetics and molecular mechanisms of hypertension has been inadequate in enabling precise diagnosis and tailored medicines for individual patients with hypertension. The genetic factors of hypertension are complicated and hypertension susceptibility gene identified so far can only explain the genetic etiology of a small number of hypertensive patients. The genetic causes of most hypertension patients are unknown. Therefore, there is a long way for us to go in elucidating the genetic basis of hypertension thoroughly.

Over the past decades, there has been a rapid increase in our understanding that the immune system plays a vital role in the regulation of renal ion channels [160]. For example, CD8^+^ T cells [161], IL-17A [162], TNF-α [163], and interferon-γ [164] have been known to be important regulators of NCC, and IL-1 receptor activates salt reabsorption in Ang II-induced hypertension via the NKCC2 in the nephron [165], which provided new insights into the targeting antihypertension therapies according to the immune mechanism of hypertension. Therefore, the immune regulation on renal ion channels might become an attractive direction for hypertension research in future.

In the present review, we discuss the progress of genetics studies on kidney ion handling genes and their interaction in blood pressure regulation, and provide a panel of genes effecting kidney sodium reabsorption. Since hypertension is a polygenic disease and there are G × G interactions between kidney sodium reabsorption-related genes, it may be more effective to detect a gene panel than to detect a single susceptibility gene for patients with hypertension. Taking into account the key role of kidney sodium reabsorption in blood pressure control, in future studies, combining all the mutations of renal sodium reabsorption-related genes for a comprehensive analysis is more helpful and powerful for clarifying the mechanisms of essential hypertension.

## Supplementary Material

Supplementary Tables S1-S3

## Abbreviations

BP: blood pressure
CCD: cortical collecting duct
CD: collecting duct
CHO: Chinese hamster ovary
CLC: chloride channel
CLC-Kb: chloride voltage-gated channel Kb
CNT: connecting tubule
DBP: diastolic blood pressure
DCT: distal convoluted tubule
EH: essential hypertension
ENaC: epithelial Na^+^ channel
KO: knockout
KS-WNK1: kidney-specific WNK1
L-WNK1: full-length WNK1
MAP: mean arterial pressure
NCC: Na^+^-Cl^−^ cotransporter
NKCC2: Na-K-2Cl cotransporter
PCT: proximal convoluted tubule
PHA 1: pseudohypoaldosteronism type 1
PHA 2: pseudohypoaldosteronism type 2
RAAS: renin-angiotensin-aldosterone system
ROMK: renal outer medullary potassium channel
SBP: systolic blood pressure
SGK1: serum- and glucocorticoid-regulated kinase 1
TAL: thick ascending limb
TALHL: thick ascending limb of Henle’s loop
TM: transmembrane
WNK: with no lysis (K) kinase
