# Supplementary material for: Kidney ion handling genes and their interaction in blood pressure control
Source: Biosci Rep. 2022 Nov 16;42(11):BSR20220977. doi: 10.1042/BSR20220977 (PMC9670246; doi:10.1042/BSR20220977)
Supplement: Supplementary Tables S1-S3 [file BSR-2022-0977_supp.zip › BSR-2022-0977_suppS1.pdf]

Supplementary Table 1 (Table S1). Renal sodium reabsorption-related genes involved in blood pressure regulation

Gene	Protein	Function	Reference
ACE	Angiotensin I converting enzyme (ACE)	This gene encodes an enzyme involved in blood pressure regulation and electrolyte balance.	[1,2]
ADD1	α-Adducin	Adducins are a family of cytoskeletal proteins encoded by three genes (α, β, and γ). Of these, α-Adducin has the closest relationship with EH, which mutations enhance the renal Na^+ reabsorption.	[3-5]
AGTR1	Angiotensin II receptor type 1 (AGTR1)	Angiotensin II is a potent vasopressor hormone and a primary regulator of aldosterone secretion. It is an important effector controlling blood pressure and volume in the cardiovascular system. It acts through at least two types of receptors. This gene encodes the type 1 receptor which is thought to mediate the major cardiovascular effects of angiotensin II.	[2]
AGTR2	Angiotensin II receptor type 2 (AGTR2)	It functions as a receptor for angiotensin II, involving in renal Na^+ reabsorption and hypertension.	[6]
Angiotensinogen (*AGT*)	Pre-angiotensinogen or angiotensinogen precursor	It is cleaved by the enzyme renin in response to lowered blood pressure, involving in RAAS, which regulates ENaC in the kidney. Mutations in this gene are associated with susceptibility to essential hypertension.	[2,7,8]
ATP1B1	Na,K-ATPase β subunit	An intrinsic oligomeric protein necessary for the maintenance of Na^+ and K^+ electrochemical gradients across the plasma membrane. Involved in multiple BP-regulating physiological processes: renal sodium reabsorption, vascular smooth-muscle-tone regulation, and cardiac muscle contraction.	[9,10]
CLCN2	Chloride voltage-gated channel 2	The transmembrane protein maintains chloride ion homeostasis in various cells, and is also expressed in adrenal glomerulosa and induces	[11]

		expression of aldosterone synthase.	
CLCNKA	Chloride voltage-gated channel Ka (CLC-Ka)	This gene is a member of the CLC family of voltage-gated chloride channels. The encoded protein requires a beta subunit called barttin to form a functional channel. It is thought to function in salt reabsorption in the kidney and potassium recycling in the inner ear. The gene is highly similar to CLCNKB, which is located 10 kb downstream from this gene.	[12]
CLCNKB	Chloride voltage-gated channel Kb (CLC-Kb)	This channel is expressed predominantly in the kidney and may be important for renal salt reabsorption.	[13-15]
CLDNs	Claudins	Claudins are integral membrane proteins that are components of the epithelial cell tight junctions, which regulate movement of solutes and ions through the paracellular space.	[16-18]
CTNNB1	β-catenin	It is the central molecule in the canonical Wnt signalling system, and linked functionally to aldosterone secretion, involving in the development of hypertension.	[19,20]
CYP11B1	Steroid 11 β-hydroxylase	This enzyme is involved in synthesis of cholesterol, and steroids.	[7]
CYP11B2	Aldosterone synthase	The enzyme has steroid 18-hydroxylase activity to synthesize aldosterone, involving in the regulation of aldosterone on renal ion channels.	[2,7,21,22]
CYP17A1	Cytochrome p450 enzyme	Cytochrome p450 enzyme mediating the first step in mineralocorticoid and glucocorticoid synthesis. Also involved in sex steroid synthesis. Mineralocorticoid and glucocorticoid participate in the regulation of renal sodium channel.	[23-26]

DRD1	dopamine receptor D1 (DRD1)	This gene encodes the D1 subtype of the dopamine receptor. The D1 subtype is the most abundant dopamine receptor in the central nervous system. DRD1 regulates NHE and Na+ and K+ ATPase, and be associated with essencial hypertension.	[27]
ENPEP	Glutamyl aminopeptidase	This protein can upregulate blood pressure by cleaving the N-terminal aspartate from angiotensin II.	[28]
GRK4	G protein-coupled receptor kinase 4	This gene encodes a member of the G protein-coupled receptor kinase subfamily of the Ser/Thr protein kinase family. The protein phosphorylates the activated forms of G protein-coupled receptors thus initiating its deactivation. This gene has been linked to both genetic and acquired hypertension mainly by regulating the activity of the dopamine receptor D1 and AT1R.	[27,29]
HSD3B1	3 beta- and steroid delta-isomerase 1	This protein plays a crucial role in the biosynthesis of aldosterone and is involved in BP regulation.	[30]
HSD11B1; HSD11B2	11β-hydroxy steroid dehydrogenase type 1; 11β-hydroxy steroid dehydrogenase type 2	They are involved in the production of glucocorticoids. Hypertension caused by the overexpression of the HSD11B gene is associated with the synthesis of a glucocorticoid-dependent angiotensinogen. A particular variation in the *HSD11B1* gene has been associated with obesity and insulin resistance in children. Mutations in the *HSD11B2* gene cause the syndrome of apparent mineralocorticoid excess and hypertension.	[31,32]
KCNJ1	ROMK	ROMK strictly regulates the secretion of potassium in CCD, controls the potassium cycle in TALHL of kidney, and exerts roles in kidney sodium reabsorption.	[33,34]

KCNJ11	Potassium inwardly rectifying channel subfamily J member 11	The protein has a greater tendency to allow potassium to flow into a cell rather than out of a cell, and it is associated with both diabetes and hypertension. KCNJ11 was identified as a target for minoxidil and diazoxide (antihypertensive medications).	[35,36]
NR3C2 (nuclear receptor subfamily 3 group C member 2)	Mineralocorticoid Receptor (MR)	MR mediates aldosterone actions on salt and water balance within restricted target cells. Defects in this gene are associated with early onset hypertension with severe exacerbation in pregnancy.	[1,37]
REN	Renin	Renin is a part of RAAS involved in blood pressure regulation, and electrolyte balance.	[38,39]
SCNN1A; *SCNN1B*; *SCNN1G*	Epithelial sodium channel α, β, γ subunits (ENaCα, ENaCβ, and ENaCγ, respectively)	ENaC is responsible for the rate-limiting reabsorption of Na^+ in the kidney.	[40-42]
SGK1	Serum/glucocorticoid regulated kinase 1 (SGK1)	This kinase activates certain K^+, Na^+, and Cl^- channels, suggesting an involvement in the regulation of renal sodium excretion.	[43-46]
SLC4A5	Na^+-HCO_3^- cotransporter, also known as NBC4; NBCe2	Sodium bicarbonate cotransporters are involved in intracellular pH regulation and electroneural or electrogenic sodium bicarbonate transport, taking part in the pathogenesis of hypertension.	[47-49]
SLC9A1 (*solute carrier family 9 member A1*)	Na^+/H^+ exchanger 1 (NHE1)	This protein is a plasma membrane transporter that is expressed in the kidney and intestine. It functions through an inward sodium ion chemical gradient to eliminate acids (protons) generated by metabolism and regulate intracellular pH.	[50]
SLC9A3(*solute carrier family 9 member A3*)	Na^+/H^+ exchanger 3 (NHE3)	A sodium-hydrogen antiporter expressed primarily by epithelial cells in the kidneys, it localizes to the apical membrane of the proximal kidney tubule, where it functions in sodium and water reabsorption and possibly calcium homeostasis.	[51]

SLC12A1	Na-K-2Cl cotransporter (NKCC2)	NKCC2 is one transporter with the strongest renal sodium reabsorption capacity and responsible for nearly 24% of total renal sodium reabsorption.	[33,52]
SLC12A3	Na^+-Cl^- cotransporter (NCC)	This cotransporter is important for electrolyte homeostasis and mediates sodium and chloride reabsorption in the DCT.	[33,53]
SLC26A4	Pendrin	It secretes bicarbonate and exchanges chloride ions in the lumen to maintain acid-base balance, participates in the reabsorption of chloride ions, regulates blood pressure and maintains body fluid homeostasis.	[54,55]
SLC26A9	solute carrier family 26 member 9	This gene is one member of a family of sulfate/anion transporter genes. The product of this gene is a highly selective chloride ion channel regulated by WNK kinases.	[56]
SGLT2	Sodium/glucose cotransporter 2	It is a sodium-dependent glucose transport proteins, and associated with Na^+ and glucose reabsorption in the kidney and BP regulation.	[57]
TGFβ	Transforming growth factor beta 1 (TGFβ)	It regulates cell proliferation, differentiation and growth, and the TGFβ pathway affects sodium handling in the kidney and associated with hypertension.	[19,58]
WNK1; WNK3; WNK4; KS-WNK1	WITH-NO-LYSINE 1, 3, 4 (WNK1, 3, and 4); Kidney-specific WNK1 (KS-WNK1)	WNKs family regulate certain K^+, and Na^+ channels, participating in the regulation of renal sodium excretion.	[59-62]

References

1 Lifton, R.P., Gharavi, A.G., Geller, D.S. (2001) Molecular mechanisms of human hypertension. *Cell* **104**, 545-556, https://doi.org/S0092-8674(01)00241-0

2 Ji, L., Cai, X., Zhang, L., Fei, L., Wang, L., Su, J., et al. (2013) Association between polymorphisms in the renin-angiotensin-aldosterone system genes and essential hypertension in the Han Chinese population. *PLoS One* **8**, e72701, https://doi.org/10.1371/journal.pone.0072701

3 Wang, L., Zheng, B., Zhao, H., Du, P., Sun, A., Hua, K., et al. (2014) alpha-Adducin gene G614T polymorphisms in essential hypertension patients with high low density lipoprotein (LDL) levels. *Indian J Med Res* **139**, 273-278

4 Kundu, A., Anand, A. (2013) Computational study of ADD1 gene polymorphism associated with hypertension. *Cell Biochem Biophys* **65**, 13-19, https://doi.org/10.1007/s12013-012-9398-2

5 Liao, X., Wang, W., Zeng, Z., Yang, Z., Dai, H., Lei, Y. (2015) Association of alpha-ADD1 Gene and Hypertension Risk: A Meta-Analysis. *Med Sci Monit* **21**, 1634-1641, https://doi.org/10.12659/MSM.893191

6 Fatima, N., Patel, S.N., Hussain, T. (2021) Angiotensin II Type 2 Receptor: A Target for Protection Against Hypertension, Metabolic Dysfunction, and Organ Remodeling. *Hypertension* **77**, 1845-1856, https://doi.org/10.1161/HYPERTENSIONAHA.120.11941

7 Lifton, R.P. (1996) Molecular genetics of human blood pressure variation. *Science* **272**, 676-680, https://doi.org/10.1126/science.272.5262.676

8 Surendran, P., Feofanova, E.V., Lahrouchi, N., Ntalla, I., Karthikeyan, S., Cook, J., et al. (2020) Discovery of rare variants associated with blood pressure regulation through meta-analysis of 1.3 million individuals. *Nat Genet* **52**, 1314-1332, https://doi.org/10.1038/s41588-020-00713-x

9 Ferrandi, M., Tripodi, G., Salardi, S., Florio, M., Modica, R., Barassi, P., et al. (1996) Renal Na,K-ATPase in genetic hypertension. *Hypertension* **28**, 1018-1025, https://doi.org/10.1161/01.hyp.28.6.1018

10 Chang, Y.P., Liu, X., Kim, J.D., Ikeda, M.A., Layton, M.R., Weder, A.B., et al. (2007) Multiple genes for essential-hypertension susceptibility on chromosome 1q. *Am J Hum Genet* **80**, 253-264, https://doi.org/10.1086/510918

11 Scholl, U.I., Stolting, G., Schewe, J., Thiel, A., Tan, H., Nelson-Williams, C., et al. (2018) CLCN2 chloride channel mutations in familial hyperaldosteronism type II. *Nat Genet* **50**, 349-354, https://doi.org/10.1038/s41588-018-0048-5

12 Chen, X., Zhou, B., Hou, X., Xing, J., Zou, S., Wu, X., et al. (2015) Associations between CLCNKA_B tag SNPs with essential hypertension and interactions between genetic and environmental factors in an island population in China. *Clin Exp Hypertens* **37**, 519-525, https://doi.org/10.3109/10641963.2015.1013124

13 Jeck, N., Waldegger, S., Lampert, A., Boehmer, C., Waldegger, P., Lang, P.A., et al. (2004) Activating mutation of the renal epithelial chloride channel ClC-Kb predisposing to hypertension. *Hypertension* **43**, 1175-1181, https://doi.org/10.1161/01.HYP.0000129824.12959.f0

14 Sile, S., Velez, D.R., Gillani, N.B., Narsia, T., Moore, J.H., George, A.L., Jr., et al. (2009) CLCNKB-T481S and essential hypertension in a Ghanaian population. *J Hypertens* **27**, 298-304, https://doi.org/10.1097/hjh.0b013e3283140c9e

15 Kokubo, Y., Tomoike, H., Tanaka, C., Banno, M., Okuda, T., Inamoto, N., et al. (2006) Association of sixty-one non-synonymous polymorphisms in forty-one hypertension candidate genes with blood pressure variation and hypertension. *Hypertens Res* **29**, 611-619, https://doi.org/10.1291/hypres.29.611

16 Varadarajan, S., Stephenson, R.E., Miller, A.L. (2019) Multiscale dynamics of tight junction remodeling. *J Cell Sci* **132**, jcs229286, https://doi.org/10.1242/jcs.229286

17 Fromm, M., Piontek, J., Rosenthal, R., Gunzel, D., Krug, S.M. (2017) Tight junctions of the proximal tubule and their channel proteins. *Pflugers Arch* **469**, 877-887, https://doi.org/10.1007/s00424-017-2001-3

18 Reyes, J.L., Lamas, M., Martin, D., del Carmen Namorado, M., Islas, S., Luna, J., et al. (2002) The renal segmental distribution of claudins changes with development. *Kidney Int* **62**, 476-487, https://doi.org/10.1046/j.1523-1755.2002.00479.x

19 Evangelou, E., Warren, H.R., Mosen-Ansorena, D., Mifsud, B., Pazoki, R., Gao, H., et al. (2018) Genetic analysis of over 1 million people identifies 535 new loci associated with blood pressure traits. *Nat Genet* **50**, 1412-1425, https://doi.org/10.1038/s41588-018-0205-x
20 Teo, A.E., Garg, S., Shaikh, L.H., Zhou, J., Karet Frankl, F.E., Gurnell, M., et al. (2015) Pregnancy, Primary Aldosteronism, and Adrenal CTNNB1 Mutations. *N Engl J Med* **373**, 1429-1436, https://doi.org/10.1056/NEJMoa1504869
21 Ji, X., Qi, H., Li, D.B., Liu, R.K., Zheng, Y., Chen, H.L., et al. (2015) Associations between human aldosterone synthase CYP11B2 (-344T/C) gene polymorphism and antihypertensive response to valsartan in Chinese patients with essential hypertension. *Int J Clin Exp Med* **8**, 1173-1177
22 Li, W., Liu, C. (2014) The -344C/T polymorphism in the CYP11B2 gene is associated with essential hypertension in the Chinese. *J Renin Angiotensin Aldosterone Syst* **15**, 150-155, https://doi.org/10.1177/1470320312466928
23 Coffman, T.M. (2011) Under pressure: the search for the essential mechanisms of hypertension. *Nat Med* **17**, 1402-1409, https://doi.org/10.1038/nm.2541
24 Mussig, K., Kaltenbach, S., Machicao, F., Maser-Gluth, C., Hartmann, M.F., Wudy, S.A., et al. (2005) 17alpha-hydroxylase/17,20-lyase deficiency caused by a novel homozygous mutation (Y27Stop) in the cytochrome CYP17 gene. *J Clin Endocrinol Metab* **90**, 4362-4365, https://doi.org/10.1210/jc.2005-0136
25 Newton-Cheh, C., Johnson, T., Gateva, V., Tobin, M.D., Bochud, M., Coin, L., et al. (2009) Genome-wide association study identifies eight loci associated with blood pressure. *Nat Genet* **41**, 666-676, https://doi.org/10.1038/ng.361
26 Xi, B., Shen, Y., Reilly, K.H., Wang, X., Mi, J. (2013) Recapitulation of four hypertension susceptibility genes (CSK, CYP17A1, MTHFR, and FGF5) in East Asians. *Metabolism* **62**, 196-203, https://doi.org/10.1016/j.metabol.2012.07.008
27 Zhang, H., Sun, Z.Q., Liu, S.S., Yang, L.N. (2016) Association between GRK4 and DRD1 gene polymorphisms and hypertension: a meta-analysis. *Clin Interv Aging* **11**, 17-27, https://doi.org/10.2147/CIA.S94510
28 Surendran, P., Drenos, F., Young, R., Warren, H., Cook, J.P., Manning, A.K., et al. (2016) Trans-ancestry meta-analyses identify rare and common variants associated with blood pressure and hypertension. *Nat Genet* **48**, 1151-1161, https://doi.org/10.1038/ng.3654
29 Yang, J., Villar, V.A., Jones, J.E., Jose, P.A., Zeng, C. (2015) G protein-coupled receptor kinase 4: role in hypertension. *Hypertension* **65**, 1148-1155, https://doi.org/10.1161/HYPERTENSIONAHA.115.05189
30 Salvi, E., Wang, Z., Rizzi, F., Gong, Y., McDonough, C.W., Padmanabhan, S., et al. (2017) Genome-Wide and Gene-Based Meta-Analyses Identify Novel Loci Influencing Blood Pressure Response to Hydrochlorothiazide. *Hypertension* **69**, 51-59, https://doi.org/10.1161/HYPERTENSIONAHA.116.08267
31 Rask, E., Walker, B.R., Soderberg, S., Livingstone, D.E., Eliasson, M., Johnson, O., et al. (2002) Tissue-specific changes in peripheral cortisol metabolism in obese women: increased adipose 11beta-hydroxysteroid dehydrogenase type 1 activity. *J Clin Endocrinol Metab* **87**, 3330-3336, https://doi.org/10.1210/jcem.87.7.8661
32 Evans, L.C., Ivy, J.R., Wyrwoll, C., McNairn, J.A., Menzies, R.I., Christensen, T.H., et al. (2016) Conditional Deletion of Hsd11b2 in the Brain Causes Salt Appetite and Hypertension. *Circulation* **133**, 1360-1370, https://doi.org/10.1161/CIRCULATIONAHA.115.019341

33 Ji, W., Foo, J.N., O'Roak, B.J., Zhao, H., Larson, M.G., Simon, D.B., et al. (2008) Rare independent mutations in renal salt handling genes contribute to blood pressure variation. *Nat Genet* **40**, 592-599, https://doi.org/10.1038/ng.118

34 Tobin, M.D., Tomaszewski, M., Braund, P.S., Hajat, C., Raleigh, S.M., Palmer, T.M., et al. (2008) Common variants in genes underlying monogenic hypertension and hypotension and blood pressure in the general population. *Hypertension* **51**, 1658-1664, https://doi.org/10.1161/hypertensionaha.108.112664

35 Barbetti, F., D'Annunzio, G. (2018) Genetic causes and treatment of neonatal diabetes and early childhood diabetes. *Best Pract Res Clin Endocrinol Metab* **32**, 575-591, https://doi.org/10.1016/j.beem.2018.06.008

36 Eales, J.M., Jiang, X., Xu, X., Saluja, S., Akbarov, A., Cano-Gamez, E., et al. (2021) Uncovering genetic mechanisms of hypertension through multi-omic analysis of the kidney. *Nat Genet* **53**, 630-637, https://doi.org/10.1038/s41588-021-00835-w

37 Geller, D.S., Farhi, A., Pinkerton, N., Fradley, M., Moritz, M., Spitzer, A., et al. (2000) Activating mineralocorticoid receptor mutation in hypertension exacerbated by pregnancy. *Science* **289**, 119-123, https://doi.org/10.1126/science.289.5476.119

38 Athimulam, S., Lazik, N., Bancos, I. (2019) Low-Renin Hypertension. *Endocrinol Metab Clin North Am* **48**, 701-715, https://doi.org/10.1016/j.ecl.2019.08.003

39 Reiter, L.M., Christensen, D.L., Gjesing, A.P. (2016) Renin angiotensinogen system gene polymorphisms and essential hypertension among people of West African descent: a systematic review. *J Hum Hypertens* **30**, 467-478, https://doi.org/10.1038/jhh.2015.114

40 Ambrosius, W.T., Bloem, L.J., Zhou, L., Rebhun, J.F., Snyder, P.M., Wagner, M.A., et al. (1999) Genetic variants in the epithelial sodium channel in relation to aldosterone and potassium excretion and risk for hypertension. *Hypertension* **34**, 631-637, https://doi.org/10.1161/01.hyp.34.4.631

41 Baker, E.H., Dong, Y.B., Sagnella, G.A., Rothwell, M., Onipinla, A.K., Markandu, N.D., et al. (1998) Association of hypertension with T594M mutation in beta subunit of epithelial sodium channels in black people resident in London. *Lancet* **351**, 1388-1392, https://doi.org/10.1016/s0140-6736(97)07306-6

42 Iwai, N., Baba, S., Mannami, T., Katsuya, T., Higaki, J., Ogihara, T., et al. (2001) Association of sodium channel gamma-subunit promoter variant with blood pressure. *Hypertension* **38**, 86-89, https://doi.org/10.1161/01.hyp.38.1.86

43 Busjahn, A., Aydin, A., Uhlmann, R., Krasko, C., Bähring, S., Szelestei, T., et al. (2002) Serum- and glucocorticoid-regulated kinase (SGK1) gene and blood pressure. *Hypertension* **40**, 256-260, https://doi.org/10.1161/01.hyp.0000030153.19366.26

44 Busjahn, A., Luft, F.C. (2003) Twin studies in the analysis of minor physiological differences between individuals. *Cell Physiol Biochem* **13**, 51-58, https://doi.org/10.1159/000070249

45 von Wowern, F., Berglund, G., Carlson, J., Månsson, H., Hedblad, B., Melander, O. (2005) Genetic variance of SGK-1 is associated with blood pressure, blood pressure change over time and strength of the insulin-diastolic blood pressure relationship. *Kidney Int* **68**, 2164-2172, https://doi.org/10.1111/j.1523-1755.2005.00672.x

46 Zhang, D., Gu, D., He, J., Hixson, J.E., Rao, D.C., Li, C., et al. (2017) Associations of the Serum/Glucocorticoid Regulated Kinase Genes With BP Changes and Hypertension Incidence: The Gensalt Study. *Am J Hypertens* **30**, 95-101, https://doi.org/10.1093/ajh/hpw122

47 Carey, R.M., Schoeffel, C.D., Gildea, J.J., Jones, J.E., McGrath, H.E., Gordon, L.N., et al. (2012) Salt sensitivity of blood pressure is associated with polymorphisms in the sodium-bicarbonate cotransporter. *Hypertension* **60**, 1359-1366, https://doi.org/10.1161/HYPERTENSIONAHA.112.196071

48 Gildea, J.J., Xu, P., Kemp, B.A., Carlson, J.M., Tran, H.T., Bigler Wang, D., et al. (2018) Sodium bicarbonate cotransporter NBCe2 gene variants increase sodium and bicarbonate transport in human renal proximal tubule cells. *PLoS One* **13**, e0189464, https://doi.org/10.1371/journal.pone.0189464

49 Manosroi, W., Williams, G.H. (2019) Genetics of Human Primary Hypertension: Focus on Hormonal Mechanisms. *Endocr Rev* **40**, 825-856, https://doi.org/10.1210/er.2018-00071

50 Navarro-Lopez, F., Coca, A., Pare, J.C., De La Sierra, A., Bosch, X., Urbano Marquez, A. (1993) Left ventricular hypertrophy in asymptomatic essential hypertension: its relationship with aldosterone and the increase in sodium-proton exchanger activity. *Eur Heart J* **14 Suppl J**, 38-41

51 Zhu, H., Sagnella, G.A., Dong, Y., Miller, M.A., Onipinla, A., Markandu, N.D., et al. (2004) Molecular variants of the sodium/hydrogen exchanger type 3 gene and essential hypertension. *J Hypertens* **22**, 1269-1275, https://doi.org/10.1097/01.hjh.0000125428.28861.11

52 Nandakumar, P., Morrison, A.C., Grove, M.L., Boerwinkle, E., Chakravarti, A. (2018) Contributions of rare coding variants in hypotension syndrome genes to population blood pressure variation. *Medicine (Baltimore)* **97**, e11865, https://doi.org/10.1097/MD.0000000000011865

53 Wang, L., Dong, C., Xi, Y.G., Su, X. (2015) Thiazide-sensitive Na+-Cl- cotransporter: genetic polymorphisms and human diseases. *Acta Biochim Biophys Sin (Shanghai)* **47**, 325-334, https://doi.org/10.1093/abbs/gmv020

54 Verlander, J.W., Hassell, K.A., Royaux, I.E., Glapion, D.M., Wang, M.E., Everett, L.A., et al. (2003) Deoxycorticosterone upregulates PDS (Slc26a4) in mouse kidney: role of pendrin in mineralocorticoid-induced hypertension. *Hypertension* **42**, 356-362, https://doi.org/10.1161/01.HYP.0000088321.67254.B7

55 Kim, B.G., Yoo, T.H., Yoo, J.E., Seo, Y.J., Jung, J., Choi, J.Y. (2017) Resistance to hypertension and high Cl(-) excretion in humans with SLC26A4 mutations. *Clin Genet* **91**, 448-452, https://doi.org/10.1111/cge.12789

56 Amlal, H., Xu, J., Barone, S., Zahedi, K., Soleimani, M. (2013) The chloride channel/transporter Slc26a9 regulates the systemic arterial pressure and renal chloride excretion. *J Mol Med (Berl)* **91**, 561-572, https://doi.org/10.1007/s00109-012-0973-1

57 Wilcox, C.S. (2020) Antihypertensive and Renal Mechanisms of SGLT2 (Sodium-Glucose Linked Transporter 2) Inhibitors. *Hypertension* **75**, 894-901, https://doi.org/10.1161/HYPERTENSIONAHA.119.11684

58 Nakao, E., Adachi, H., Enomoto, M., Fukami, A., Kumagai, E., Nakamura, S., et al. (2017) Elevated Plasma Transforming Growth Factor beta1 Levels Predict the Development of Hypertension in Normotensives: The 14-Year Follow-Up Study. *Am J Hypertens* **30**, 808-814, https://doi.org/10.1093/ajh/hpx053

59 Shi, R., Li, J., He, J., Meng, Q., Qian, Z., Shi, D., et al. (2018) Association of with-no-lysine kinase 1 and Serine/Threonine kinase 39 gene polymorphisms and haplotypes with essential hypertension in Tibetans. *Environ Mol Mutagen* **59**, 151-160, https://doi.org/10.1002/em.22140

60 Newhouse, S., Farrall, M., Wallace, C., Hoti, M., Burke, B., Howard, P., et al. (2009) Polymorphisms in the WNK1 gene are associated with blood pressure variation and urinary potassium excretion.

PLoS One **4**, e5003, https://doi.org/10.1371/journal.pone.0005003

61 Ghodsian, N., Ismail, P., Ahmadloo, S., Heidari, F., Haghvirdizadeh, P., Ataollahi Eshkoor, S., et al. (2016) Novel Association of WNK4 Gene, Ala589Ser Polymorphism in Essential Hypertension, and Type 2 Diabetes Mellitus in Malaysia. *J Diabetes Res* **2016**, 8219543, https://doi.org/10.1155/2016/8219543

62 Osada, Y., Miyauchi, R., Goda, T., Kasezawa, N., Horiike, H., Iida, M., et al. (2009) Variations in the WNK1 gene modulates the effect of dietary intake of sodium and potassium on blood pressure determination. *J Hum Genet* **54**, 474-478, https://doi.org/10.1038/jhg.2009.64
