# Supplementary material for: Kidney ion handling genes and their interaction in blood pressure control
Source: Biosci Rep. 2022 Nov 16;42(11):BSR20220977. doi: 10.1042/BSR20220977 (PMC9670246; doi:10.1042/BSR20220977)
Supplement: Supplementary Tables S1-S3 [file BSR-2022-0977_supp.zip › BSR-2022-0977_suppS2.pdf]

Supplementary Table 2 (Table S2). The distribution of NCC, NKCC2, ENaCs, Pendrin, WNKs and SGK1 in the kidney.

Genes	Proteins or Their Subunits	Distribution in the Kidney
SCNN1A; SCNN1B; *SCNN1G*	ENaC α (Epithelial sodium channel α subunit); ENaC β (Epithelial sodium channel β subunit); ENaC γ (Epithelial sodium channel γ subunit)	CNT and CD
SLC12A3	NCC (Na^+- Cl^- cotransportor)	The apical membrane of DCT cells
SLC12A1	NKCC2 (Na^+-K^+-2Cl^- cotransportor)	The apical membrane of TALHL
KCNJ1	ROMK (renal outer medullary potassium channel)	CCD and TALH
SLC26A4 or PDS	Pendrin (Cl^-/HCO_3^- exchanger)	Mainly distributed on the membrane of the free edge of non-A- and non-A- type wedge cells, and the cytoplasmic vesicles of the B-type wedge cells in CCD, CNT and DCT
CLCNKB	CLC-Kb	Henle's loop, DCT and CCD
PRKWNK1	WNK1	DCT and CD
PRKWNK3	WNK3	predominantly at intercellular junctions, with highest expression in PCT and TAL, and lower expression in DCT and CD
PRKWNK4	WNK4	DCT and CD
SGK1	SGK1	renal tubules
